# Network-based integrated analysis of omics data reveal novel players of TGF-β1-induced EMT in human peritoneal mesothelial cells

**DOI:** 10.1038/s41598-018-37101-9

**Published:** 2019-02-06

**Authors:** Soo Min Han, Hye-Myung Ryu, Jinjoo Suh, Kong-Joo Lee, Soon-Youn Choi, Sangdun Choi, Yong-Lim Kim, Joo Young Huh, Hunjoo Ha

**Affiliations:** 1 Graduate School of Pharmaceutical Sciences, College of Pharmacy, Ewha Womans University, Seoul, Republic of Korea; 2 Department of Pharmacology, Yonsei University College of Medicine, Seoul, Republic of Korea; 3 Department of Internal Medicine, School of Medicine, Kyungpook National University, Seoul, Republic of Korea; 4 Department of Molecular Science and Technology, Ajou University, Suwon, Republic of Korea; 5 College of Pharmacy, Chonnam National University, Gwangju, Republic of Korea

## Abstract

Long-term peritoneal dialysis is associated with progressive fibrosis of the peritoneum. Epithelial-mesenchymal transition (EMT) of mesothelial cells is an important mechanism involved in peritoneal fibrosis, and TGF-β1 is considered central in this process. However, targeting currently known TGF-β1-associated pathways has not proven effective to date. Therefore, there are still gaps in understanding the mechanisms underlying TGF-β1-associated EMT and peritoneal fibrosis. We conducted network-based integrated analysis of transcriptomic and proteomic data to systemically characterize the molecular signature of TGF-β1-stimulated human peritoneal mesothelial cells (HPMCs). To increase the power of the data, multiple expression datasets of TGF-β1-stimulated human cells were employed, and extended based on a human functional gene network. Dense network sub-modules enriched with differentially expressed genes by TGF-β1 stimulation were prioritized and genes of interest were selected for functional analysis in HPMCs. Through integrated analysis, ECM constituents and oxidative stress-related genes were shown to be the top-ranked genes as expected. Among top-ranked sub-modules, *TNFAIP6*, *ZC3H12A*, and *NNT* were validated in HPMCs to be involved in regulation of E-cadherin, ZO-1, fibronectin, and αSMA expression. The present data shows the validity of network-based integrated analysis in discovery of novel players in TGF-β1-induced EMT in peritoneal mesothelial cells, which may serve as new prognostic markers and therapeutic targets for peritoneal dialysis patients.

## Introduction

Peritoneal dialysis (PD) is an important renal replacement therapy in end-stage renal disease^1,2^. During PD, the peritoneal membrane acts as a permeable barrier in which ultrafiltration and diffusion takes place^3^. Continual exposure to PD can result in acute and chronic inflammation and injury of the peritoneal membrane^4^. Consequently, the peritoneum undergoes progressive fibrosis, angiogenesis, and vasculopathy, which ultimately can lead to discontinuation of PD^5^.

The peritoneal membrane is composed of a monolayer of mesothelial cells, and a key role in the induction of peritoneal fibrosis during exposure to PD fluids is played by epithelial to mesenchymal transition (EMT) of the mesothelial cells (MCs), named more properly mesothelial to mesenchymal transition (MMT)^6^. In human peritoneal mesothelial cells (HPMCs), a progressive loss of epithelial morphology and acquisition of a fibroblast-like phenotype (i.e., EMT) has been demonstrated from early through later stages of PD^5,7,8^. Among many mediators, TGF-β1 is central to the process of EMT and peritoneal fibrosis^9–11^. Previous studies have reported that TGF-β1 levels in PD fluids correlate with deterioration of peritoneal membrane in dialysis patients and that TGF-β1 blocking peptides preserve the peritoneal membrane from PD fluid-induced damage in mice^10,12^. Based on these findings, blockade of TGF-β1 has been regarded as an attractive therapeutic target for treatment of peritoneal fibrosis. Studies so far have found that TGF-β1-Smad pathway as well as Smad-independent MEK/ERK1/2 pathway are responsible for the progression of EMT and fibrosis in PD^13–16^. However, due to the diversity of the role of TGF-β1 in regulation of physiology, directly targeting TGF-β1 or previously known pathways have not proven successful for the development of effective therapeutics in treatment or prevention of peritoneal fibrosis. Therefore, there is still a need for discovery of a novel and more specific downstream targets involved in the TGF-β1-associated EMT and peritoneal fibrosis.

The complexity of biological processes that underlie diseases makes individual biological experiments inadequate for full explanation. As an alternative, bioinformatics enables systematic analysis and provides new insights into complicated biological networks by exploring multiple molecular components simultaneously^17^. Data integration driven by omics studies and access to online repositories and web-based algorithms has made it possible to identify biomarkers of prognostic and therapeutic value^18–20^. Due to the transient nature of the EMT process^21^, bioinformatics approaches have proven valuable adding statistical rigor to studies that would otherwise lack sufficient power to reach compelling conclusions. However, there are limited reports where peritoneal membrane associated EMT has been studied by high-throughput data analysis.

In this study we conducted microarray- and proteomics-based differential expression analysis of genes and proteins to characterize the molecular signature of TGF-β1-stimulated human peritoneal mesothelial cells (HPMCs). Then we expanded the data by integrating gene expression profiles of multiple datasets of TGF-β1-stimulated human cells. Under the assumption that essential biological modules responsible for TGF-β1-mediated EMT and fibrosis would be represented as core network modules of integrated datasets, gene co-expression profiles were utilized to construct a functional gene network (EMT-network) and then dense network clusters were extracted by topological analysis. We prioritized network clusters with statistical enrichment of genes differentially expressed by TGF-β1 treatment. Among the genes constructing these clusters, we have selected genes with unknown significance in development of EMT and peritoneal fibrosis in HPMCs as candidate novel players. To provide a causal link of the candidate genes with EMT in peritoneal cells, we evaluated the effect of genetic modulation of the target genes on TGF-β1-regulated epithelial and mesenchymal markers in HPMCs.

## Results

### Effect of TGF-β1 on expression profiles of genes and proteins in HPMCs

**Figure 1:**
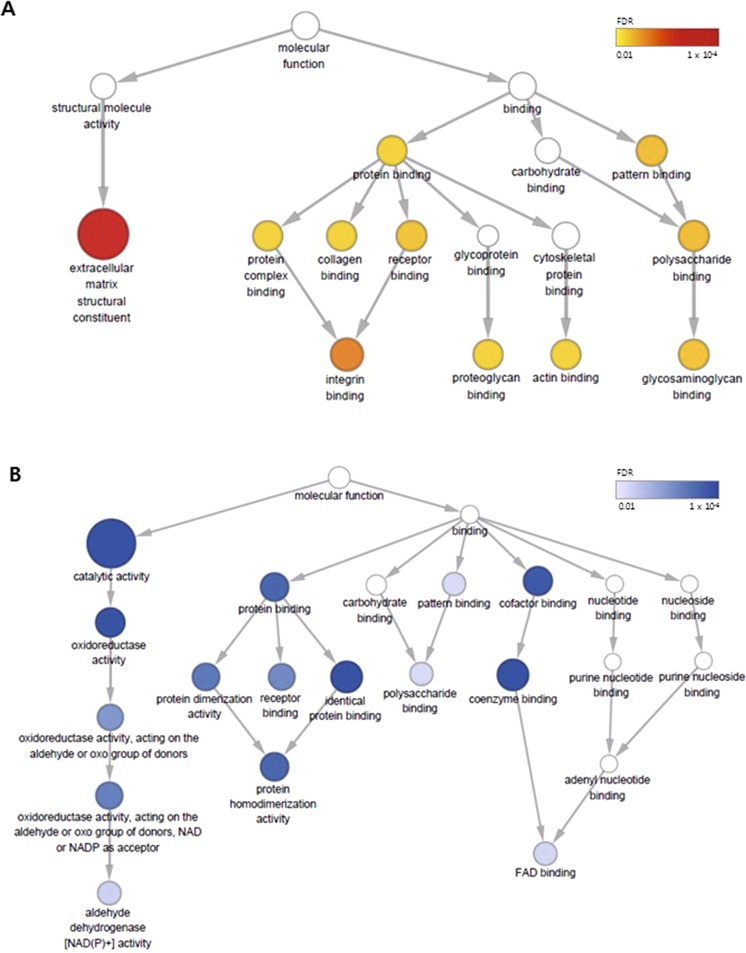
Gene ontology (GO) analysis of molecular function (MF) for DEGs by TGF-β1-stimulation in HPMCs. (**A**) The hierarchical tree of GO MF terms overrepresented by up-regulated genes (FDR < 0.01). (**B**) The hierarchical tree of GO MF terms by down-regulated genes (FDR < 0.01). Node colors and size represent the statistical significance of functional enrichment of the corresponding GO MF terms, drawn using BinGo.

A total of 1137 transcripts were differentially expressed in TGF-β1-stimulated HPMCs with a fold change above 1.5, compared to untreated HPMCs (Supplementary Table S1). Gene ontology (GO) analysis was conducted to identify significant molecular function (MF) overrepresented by DEGs (Fig. 1). The most significant GO MF term overrepresented by up-regulated genes was extracellular matrix (ECM) structural constituent, which plays a central role in EMT (Fig. 1A). Other significantly enriched GO MF terms were various binding terms, including integrin binding and collagen binding. This signature of enrichment in ECM function and organization was more detailed by the reactome pathways analysis of up-regulated genes (Supplementary Fig. S1). For down-regulated genes, catalytic activity was the most significantly enriched GO MF term, which comprised several terms related to oxidoreductase activity at lower levels (Fig. 1B). This implicated the involvement of oxidation-reduction pathways in TGF-β1-associated EMT and peritoneal fibrosis. Interestingly, other significantly enriched GO MF terms by down-regulated genes were also various binding terms at lower levels such as coenzyme binding, which showed different signatures compared to GO MF terms highlighted by up-regulated genes.

Along with transcriptomic analysis, proteomic profiles were analyzed in HPMCs treated with TGF-β1 for 48 and 96 h (Supplementary Fig. S2, and Supplementary Table S2). In alignment with the GO analysis of DEGs, the functional classification of differentially expressed proteins (DEPs) showed that structural activity, oxidoreductase activity and binding activity related proteins were essential in TGF-β1-associated EMT (Supplementary Fig. S3A). To gain further insights, 15 DEPs were chosen based on up- or down-regulation with a threshold of 1.5 fold compared to controls and mapped to the hierarchical tree of enriched GO MF terms (Supplementary Fig. S3B). Accordingly, DEPs with different changes in direction were mapped together in the same GO MF terms of structural molecular activity, protein binding and oxidoreductase activity. This suggests complex and dynamic interactions between molecular players in TGF-β1-associated EMT and the necessity of systematic investigations of altered molecular signatures induced by TGF-β1 treatment in HPMCs.

### Network-based integrated analysis of DEGs in TGF-β1-induced EMT

**Figure 2:**
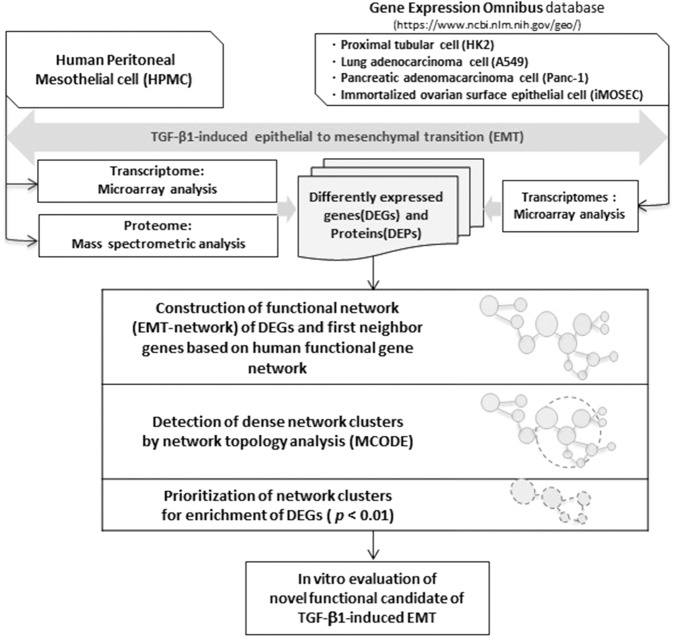
A workflow of network-based integrated analysis to identify novel molecular players of TGF-β1-induced EMT in HPMCs.

We next obtained four datasets from the Gene Expression Omnibus (GEO) database, where TGF-β1 was commonly used to induce EMT *in vitro*, to additionally analyze for DEGs under the assumption that there exists a commonly shared core module underlying the EMT process (Table 1 and Fig. 2). Among total of 2128 up-regulated and 2420 down-regulated genes from pooled data, 246 were commonly up-regulated DEGs and 347 were commonly down-regulated DEGs in at least two datasets (Supplementary Table S3, Supplementary Fig. S4). These genes were utilized as a seed set to construct the ‘EMT-network’. DEPs were also added to the seed set. The initial network was extended to first neighbors of seed genes based on interaction evidence obtained from the human functional gene network (HumanNet, http://www.functionalnet.org/humannet/)^22^. By extending the network to first neighbors of seed genes, we expected to include undisclosed relations between DEGs by direct or indirect connections, thereby facilitating identification of core network modules by topological analysis in a comprehensive gene network. The resulting network consisted of 9,654 nodes and 388,756 linkages, including DEGs and genes with functional association with the DEGs.

**Table 1:** Characteristics of microarray datasets retrieved from GEO public database.

GEO accession number	Cell line	Treating agent	Treating time	Platform	Context	Subset (samples)
GSE20247	HK2 human Proximal tubular cells	2 ng/ml of TGF-β1	48 hours	GPL6884: Illumina HumanWG-6 v3.0 expression beadchip	EMT in diabetic nephropathy [ref.^56^]	3 pairs (Control: GSM507414, GSM507415, GSM507416, Treated: GSM507420, GSM507421, GSM507422)
GSE17708	A549 lung adenocarcinoma cells	5 ng/ml of TGF-β1	24 hours	GPL570: Affymetrix Human Genome U133 Plus 2.0 Array	EMT in cancer [ref.^57^]	3 pairs (Control: GSM442026, GSM442027, GSM442028, Treated: GSM442046, GSM442047, GSM442048)
GSE23952	Panc-1 pancreatic adenocarcinoma cells	5 ng/ml of TGF-β1	48 hours	GPL570: Affymetrix Human Genome U133 Plus 2.0 Array	EMT in cancer [ref.^58^]	3 pairs (Control: GSM590166, GSM590167, GSM590168, Treated: GSM590169, GSM590170, GSM590171)
GSE6653	Immortalized ovarian surface epithelial cells (iMOSEC)	10 ng/ml of TGF-β1	12 hours	GPL570: Affymetrix Human Genome U133 Plus 2.0 Array	TGF-β/SMAD regulatory modules [ref.^59^]	2 pairs (Control: GSM154124, GSM154125, Treated: GSM154130, GSM154131)

### Detection of topologically dense network clusters enriched with DEGs

**Figure 3:**
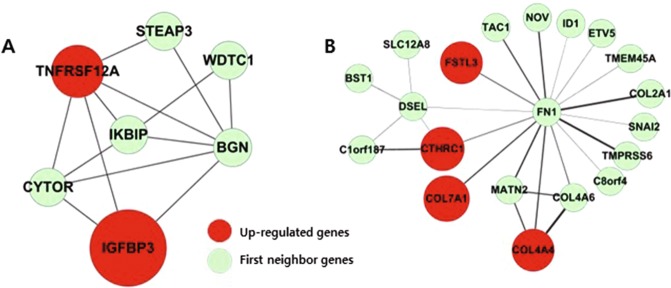
Network clusters representing EMT process, including genes with known association to EMT. Top two network clusters with the most significant enrichment for up-regulated genes. (**A**) Module I *(P* = 0.0004), (**B**) Module II *(P* = 0.001).

Next, dense network clusters were selected based on network topology analysis and prioritized by the enrichment of DEGs to reveal novel molecular players in TGF-β1-induced EMT. By applying MCODE algorithm, we obtained 861 topologically dense network clusters from 10 independent analytical runs with different conditions. To determine enriched network clusters representing significant numbers of DEGs, we performed hypergeometric tests for each detected network cluster. We assumed that network sub-modules enriched with DEGs would represent the essential functional module underlying TGF-β1 induced EMT. A total of nine network sub-modules showed statistically significant enrichment (*P* < 0.01) with either elevated or diminished expression (Table 2). In the top ranked network (module I), DEGs such as *TNFRSF12A* (also known as *FN14*) and *IGFBP3* were included which are known to enhance TGF-β1-mediated EMT (Fig. 3A)^23–25^. The first neighbor gene in this sub-module included *CYTOR*, also known as *Linc00152*, which was also previously reported as a regulator of EMT^26,27^. Various types of collagens comprised the second highly ranked network (module II) and other collagens and fibronectin were also included as first neighbor genes (Fig. 3B). This confirmed that network-based integrated analysis of multiple datasets can effectively detect core functional sub-modules of TGF-β1-inducd EMT.

**Table 2:** Details of dense network sub-modules with enrichment of DEGs (p < 0.01).

Module	Module enrichment	*P* value (hypergeometic test)	DEGs in sub-network	Differential expressions in each cell lines					Nodes in sub-network
				HPMC	HK2	A549	Panc-1	iMOSEC	
I	up-DEGs	4.30 × 10^−4^	*IKBIP*, *IGFBP3*, *TNFRSF12A*	√		√	√	√	*BGN*, *CYTOR*, *STEAP3*, *WDTC1*
II	up-DEGs	0.001	*COL4A4*, *COL7A1*, *CTHRC1*, *FSTL3*	√		√	√	√	*BST1*, *COL2A1*, *COL4A6*, *DRAXIN*, *DSEL*, *ETV5*, *FN1*, *ID1*, *MATN2*, *NOV*, *SNAI2*, *TAC1*, *TMEM45A*, *C8ORF4*, *SLC12A8*, *TMPRSS6*
III	down-DEGs	0.003	***NNT***, *GMPR*, *SELENBP1*	√		√	√		*CDH2*, *DECR1*, *FABP7*, *IMMT*, *MDH1*, *RNF103*, *VPS24*
IV	up-DEGs	0.004	*CYR61*, *JMJD1C*, *LRRC8C*			√	√		*AGXT*, *FSCN1*, *GPHA2*, *HR*, *JMJD6*, *KDM6B*, *MT1X*, *SLC3A1*, *SLC7A5*, *SLC7A8*, *SOSTDC1*
V	up-DEGs	0.005	***TNFAIP6***, ***ZC3H12A***, *SOD2*	√		√	√	√	*IL1RN*, *PDE4B*, *SOD2*
VI	down-DEGs	0.007	*ABCC6*, *LGR4*			√	√		*ABCC6P2*, *DMKN*, *RNF180*
VII	down-DEGs	0.007	*TBL1X*, *TBL1XR1*	√		√	√		*LOC285074*, *MTO1*
VIII	down-DEGs	0.007	*DHRS13*, *DHRS3*		√	√	√	√	*HSD11B1*, *HSD17B8*
IX	up-DEGs	0.008	*DSG2*, *LBH*			√	√	√	*DSC1*, *DSC2*, *IKZF5*, *USF3*

### Discovery of novel candidates downstream of TGF-β1 in EMT from dense network clusters enriched with DEGs

**Figure 4:**
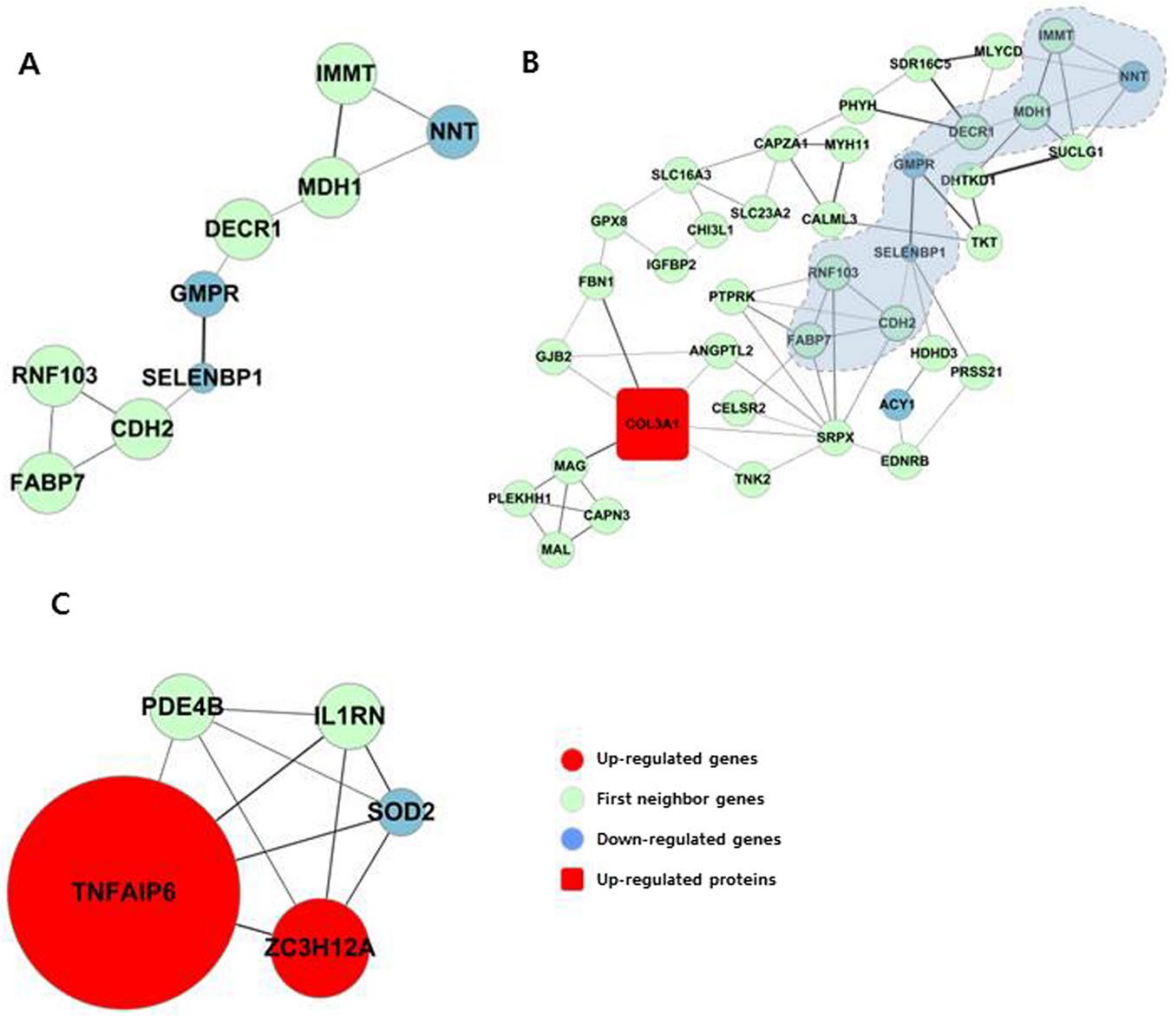
Dense network clusters significantly enriched for DEGs reveal novel molecular players in TGF-β1-associated EMT. (**A**) Most significantly enriched sub-module that includes down-regulated genes, *NNT*, *GMPR* and *SELENBP1* (Module III, *P* = 0.003). (**B**) Expanded network showing significantly enriched down-regulated genes (*P* = 0.043). The sub-network shows linkage with the up-regulated protein COL3A1, one of the conventional EMT markers. The core module enriched more significantly was blue-shadowed. (**C**) Significantly enriched sub-module including up-regulated genes (*TNFAIP6* and *ZC3H12A*) and a down-regulated gene (*SOD2*). The sub-network was ranked 5th among significantly enriched sub-modules (Module V, *P* = 0.005).

Besides the top two network sub-modules, topologically dense network clusters enriched with DEGs revealed novel genes that have not yet been shown to be associated with EMT in peritoneal membranes (Table 2). Among these, we focused on sub-networks having DEGs from HPMCs in a cell-specific manner. The third most significantly enriched sub-network (module III) included down-regulated DEGs, such as *NNT*, *SELENBP1*, and *GMPR* (*P* = 0.003, Fig. 4A). Of these, nicotinamide nucleotide transhydrogenase encoded by *NNT* is known to reduce redox potential and reactive oxygen species (ROS) levels^28^. Interestingly, the dense network cluster expanded from module III was comprised of *COL3A1*, one of DEPs with upward changes, and downregulated gene, *ACY1* (*P* = 0.043, Fig. 4B). In another enriched network sub-module (V), upregulated genes, *TNFAIP6* and *ZC3H12A*, and downregulated gene, *SOD2*, were directly connected (*P* = 0.005, Fig. 4C). Whereas the role of *TNFAIP6* and *ZC3H12A* in peritoneal fibrosis have not been reported yet, superoxide dismutase 2 encoded by *SOD2* is a mitochondrial antioxidant previously known to play a role in protection against peritoneal fibrosis^29^. These results imply a close link between regulation of oxidative stress and EMT process and suggests important roles for these novel molecular targets in regulation of TGF-β1-induced EMT. Other enriched sub-networks were mostly comprised of DEGs and first neighbor genes whose roles in EMT have not been clearly recognized (Table 2). Next, we further tested *NNT*, *TNFAIP6* and *ZC3H12A in vitro* as novel candidates downstream of TGF-β1 in EMT.

### Evaluation of therapeutic potential of novel molecular players in HPMCs

**Figure 5:**
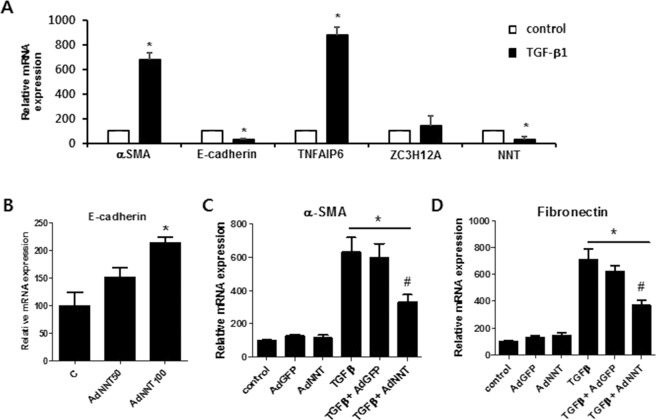
*In vitro* evaluation of the expression of novel target genes in TGF-β1-induced EMT and the effect of NNT in modulation of TGF-β1-regulated EMT markers in HPMCs. (**A**) The gene expression of EMT markers and target genes *TNFAIP6*, *ZC3H12A*, and *NNT* after treatment of 2 ng/mL TGF-β1 for 48 h. (**B**–**D**) Changes in E-cadherin and TGF-β1-induced αSMA and fibronectin gene expression after *NNT* overexpression. Values are means ± SE of 4 experiments. *p < 0.05 vs control, ^#^*p* < 0.05 vs TGFβ.

**Figure 6:**
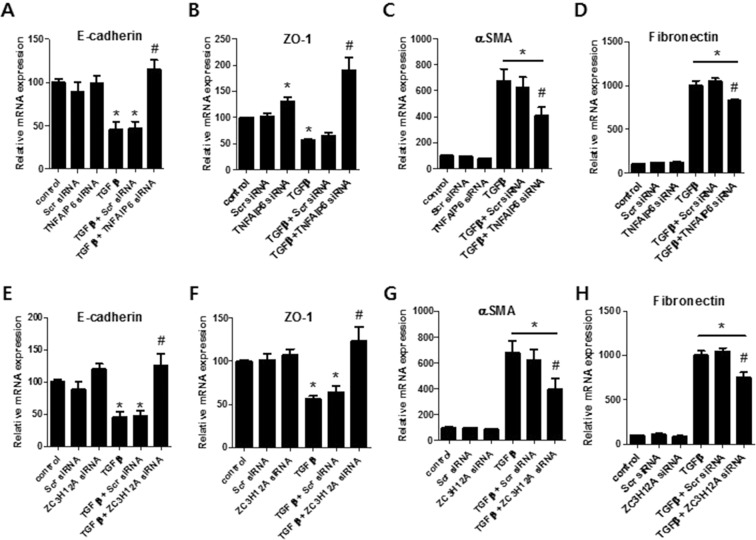
*In vitro* evaluation of the effect of TNFAIP6 and ZC3H12A in modulation of TGF-β1-regulated EMT markers.in HPMCs. (**A**–**D**) Changes in TGF-β1-induced E-cadherin, ZO-1, αSMA and fibronectin gene expression after *TNFAIP6* siRNA transfection. (**E**–**H**) Changes in TGF-β1-induced E-cadherin, ZO-1, αSMA and fibronectin gene expression after *ZC3H12A* siRNA transfection. Values are means ± SE of 4 experiments. *p < 0.05 vs control, ^#^*p* < 0.05 vs TGFβ.

Since the EMT-network was constructed based on a meta-analysis of the transcriptomes and proteomes of various types of cells, we tested whether the expression profiles of our novel candidates are regulated specifically in HPMCs by TGF-β1 stimulation. Under conditions in which TGF-β1 significantly induced alpha smooth muscle actin (αSMA) expression and inhibited E-cadherin, mRNA expressions of *TNFAIP6* and *ZC3H12A* were upregulated and mRNA expression of *NNT* was significantly downregulated (Fig. 5A), confirming results from the integrated network analysis. Next, to evaluate whether the selected targets are involved in mediating the downstream mechanisms of TGF-β1, we conducted gain-of-function and loss-of-function experiments in HPMCs. Adenoviral overexpression of *NNT* (Supplementary Fig. S5A) resulted in upregulation of E-cadherin and downregulation of TGF-β1-induced αSMA and fibronectin mRNA expression in HPMCs (Fig. 5B–D). In addition, knockdown of both *TNFAIP6* and *ZC3H12A* (Supplementary Fig. S5B,C) reversed the reduction in E-cadherin and ZO-1 and increase in αSMA and fibronectin mRNA expression under TGF-β1 treatment of HPMCs (Fig. 6). These data imply that the novel target genes identified through integrated network analysis are regulated by TGF-β1 and furthermore, these genes have the potential to regulate TGF-β1-induced EMT in peritoneal cells.

## Discussion

In this study, we have been able to reveal novel molecular players in TGF-β1-induced EMT as well as potential target candidates for treatment of EMT-associated peritoneal fibrosis through an integrated analysis. Among discovered network clusters, significantly enriched DEGs revealed novel targets such as *TNFAIP6*, *ZC3H12A*, and *NNT* which were confirmed in HPMCs *in vitro* to be associated with TGF-β1-induced EMT.

Despite recent controversies on EMT as a source of myofibroblast in the kidney and liver^30,31^, prior studies have implicated EMT as a potential therapeutic target for preventing peritoneal fibrosis^3,6,32^. We first focused on the functional enrichment of differentially expressed genes and proteins by TGF-β1 treatment in HPMCs. As expected, terms such as ECM structural constituent and collagen binding were significantly upregulated. On the other hand, oxidoreductase activity was among the most significantly downregulated molecular functions. Oxidative stress is known to be a major determinant of high glucose or TGF-β1-induced EMT of peritoneal mesothelial cells^33^. Previous reports have observed that anti-oxidants, including N-acetyl cysteine, apocynin, and mitoQ, alleviated TGF-β1-induced EMT in HPMCs^34^. Hence, through gene ontology analysis and gene set enrichment analysis we were able to generate a comprehensive view of the functional changes in TGF-β1-treated HPMCs.

We next integrated the transcriptome data available from public database to our transcriptome and proteome data in HPMCs to increase the power of the data. We focused on two goals; (1) confirmation of the validity of the meta-analysis and (2) discovery of novel molecular players associated with TGF-β1-induced EMT. Rapid advances in statistical and computational methods for data integration has enabled identification of genes from a systemic perspective^35–37^. However, systematic approaches often result in large lists of candidate genes and inevitably need efficient prioritization strategies for functional evaluation of the candidate genes. Network analysis can be one of these prioritization methods^22,38,39^. Although the network analysis does not reveal undisclosed relationships between genes, we assumed that the network analysis would reveal the relationships between DEGs in a functional manner, which could be the core modules of EMT process. To enable this, we used differently co-expressed genes as the seed set to construct the initial network (EMT-network) and then extended to the first neighbors to investigate direct and indirect links between these genes. Based on the network, we searched dense network clusters by topological analysis and prioritized network clusters enriched in DEGs from transcriptome data.

Among the significantly enriched network sub-modules, the top two highly ranked networks included known factors associated with EMT, such as *TNFRSF12A*, *IGFBP3*, and various types of collagens (Fig. 4). Of note, knockdown of *TNFRSF12A* was shown to inhibit hepatocellular carcinoma cell proliferation and migration^40^, and *IGFBP3* was reported as being involved in TGF-β1-mediated EMT in human esophageal cells and prostate cancer cells^25,41^. These results confirm that a meta-analysis based on network topology successfully retrieved networks of genes associated with the EMT pathway.

Of major interest were other network sub-modules that included novel genes that appear to play an important role in regulation of TGF-β1-induced EMT, including *NNT*, *GMPR*, *SELENBP1*, *TNFAIP6*, and *ZC3H12A*. Downregulated DEGs included in the network such as *NNT*, *GMPR*, and *SELENBP1* are involved in oxidation-reduction processes and development of carcinoma^28,42,43^, but whether these genes are key factors in linking oxidative stress to EMT process is currently unknown. As the expanded version of this network included *COL3A1* as an upregulated DEG, it is likely that downregulation of these genes are related to upregulation of ECM coding genes. In support of this, it has recently been reported that selenium has a protective effect in lipopolysaccharide-stimulated peritoneal mesothelial cells through inhibition of EMT^44^. Tumor necrosis factor-stimulated gene 6 protein (TSG-6) encoded by *TNFAIP6* has been reported to play a central role in controlling EMT in proximal tubular epithelial cells^45^, but has never been referenced in relation to HPMCs. Recently, TSG-6 was also highlighted as a therapeutic protein abundantly produced by mesenchymal stem cells to prevent fibrotic progress^46^. *ZC3H12A*, also known as *MCPIP1*, has been reported as an RNase related to immune responses^47^. Interestingly, *ZC3H12A* has been recently reported to contribute to clear cell renal cell carcinoma^48,49^. The fact that upregulation of *TNFAIP6* and *ZC3H12A* are directly associated with downregulation of *SOD2* further supports the strong relationship between oxidative stress and EMT progression.

In a subsequent experiment where we validated the expression and function of novel candidates in HPMCs by genetic manipulation, gene silencing of upregulated DEGs, *TNFAIP6* and *ZC3H12A* and overexpression of downregulated DEG, *NNT* commonly reversed biomarkers of EMT, including the loss of the epithelial adhesion protein E-cadherin and upregulation of the mesenchymal marker αSMA under TGF-β1 treatment. Whether these genes are sufficient to block TGF-β1-induced peritoneal fibrosis needs to be further evaluated by *in vivo* experiments. It is interesting to note that the gene ontology analysis and network topology analysis both showed a close association of oxidative stress and EMT in TGF-β1-treated cells. Therefore, it should also be examined whether *TNFAIP6* and *ZC3H12A* additionally exert anti-oxidative effects in peritoneal membrane.

There have been numerous attempts to identify TGF-β1-induced EMT-associated gene expression signatures^50–53^. However, to our knowledge, this is the first to integrate the EMT-related gene network in HPMCs and cancer cells. The advantage of meta-analysis is that commonly regulated transcriptomic networks can be identified, through which novel targets can serve both as a candidate for cancer treatment, as well as for other diseases where EMT is involved. Integration of multiple data sets and network-based analysis successfully overcame the limitation of initial experiments performed in HPMCs due to small data sets. Top ranked network clusters showed DEGs and genes with known functional significance in EMT, which confirmed the validity of the network analysis. The DEGs with unknown significance in ranked network clusters were further selected for evaluation *in vitro*, to confirm their biological relevance in the EMT process in peritoneal cells, which is necessary to strengthen the utility of network-based integrated analysis. Although we have validated the role of specific novel targets in alleviating the EMT biomarkers in HPMCs, it is crucial that these genes are examined at the *in vivo* level for their phenotypic effect, either through pharmacological or genetic means.

In conclusion, an omics based integrated approach has provided an extensive view of TGF-β1-induced EMT in peritoneal mesothelial cells. This work not only provides the methodological benefit but also shed new light onto the transcriptional landscape of EMT. The functional network of DEGs was detected by network topology analysis, which led to discovery of novel molecular players in TGF-β1-induced EMT. Since targeting the classical TGF-β1-Smad pathway has not proven efficacious for treatment or prevention of peritoneal fibrosis, the targets identified here may serve in development of new prognostic tools and therapeutics for peritoneal dialysis patients.

## Methods

### Cell culture

Human omentum tissue was obtained from consenting patients undergoing abdominal surgery under institutional review board (IRB)-approved protocol (Kyungpook National University IRB no. 2016-04-002-001). A written informed-consent was obtained from all subjects before the study. All experiments were performed in accordance with relevant guidelines and regulations. HPMCs were isolated, cultured, and characterized as described previously^54^. Briefly, HPMCs were isolated by enzymatic disaggregation with 0.25% trypsin–EDTA, and then cells were maintained in Medium 199 (M199) supplemented with 20% fetal bovine serum (FBS) at 37 °C in a humidified atmosphere of 5% CO2 and 95% air. For experiments, cultured HPMCs were seeded at 80% confluence in M199 containing 20% FBS for 24 h and then incubated with serum-free medium to arrest and synchronize cell growth. The cells were then exposed to recombinant human TGF-β1 (R&D Systems, Inc., Minneapolis, MN) for various times and concentrations.

### Microarray analysis of TGF-β1 treated HPMCs and meta-analysis of transcriptomes

Total RNA was extracted from HPMCs cultured under 1 ng/mL TGF-β1 for 24 h using Trizol (Invitrogen) according to standard methods. Four different HPMC samples for each group (control and TGF-β1-treated) were pooled onto one chip. For microarray analysis, 0.5 μg of total RNA was used to make biotin-labeled cRNA using the Ambion Illumina cRNA amplification and labeling kit (Ambion, Austin, TX, USA) was then column purified and the quality verified by Agilent 2100 Bioanalyzer (Palo Alto, CA, USA) prior to hybridization. Biotin-labeled cRNA was labeled with Cy3 (Amersham, Piscataway, NJ, USA) and hybridized onto a HumanRef-8 24 K Expression Array Bead Chip (Illumina, San Diego, CA, USA). The arrays were then scanned by an Illumina Bead Station laser scanning imaging system. The data were analyzed using Genespring software from Agilent after normalization. Filtered genes by diffscore value (−13 < P < 13) and fold change (FC > 1.5) were clustered using K-means and heat map clustering algorithm of Avadis. The gene ontology terms enriched in TGF-β1 treated HPMCs were identified and visualized using BiNGO v.3.0.3 plugin in Cytoscape 3.3.0^55^. Microarray data is deposited in Gene Expression Omnibus (GEO) as GSE121372 at https://www.ncbi.nlm.nih.gov/geo/query/acc.cgi?acc = GSE121372.

For meta-analysis of multiple datasets of TGF-β1-stimulated human cells, we retrieved four additional microarray datasets from GEO (http:// www.ncbi.nlm.nih.gov/geo/). Different human cells were treated with TGF-β1 in each experimental setting (Table 1)^56–59^. Raw intensity values from each of the datasets were preprocessed by log_2_ transformation. Differentially expressed genes (DEGs) between TGF-β1 treated cells and corresponding controls for each dataset were extracted with fold-change and false discovery rate (FDR) thresholds of 1.5-fold and 5%, respectively. For selection of DEGs, we applied Significance Analysis of Microarrays (SAM) by using the R package ‘samr’ (http:// statweb.stanford.edu/~tibs/SAM/). Because we did not pool the different datasets, batch-correction was not particularly applied for the analysis. Venn diagrams were drawn using web-based tool to show how many of the measured gene expression profiles overlapped between the different studies (http://bioinformatics.psb.ugent.be/webtools/Venn/).

### Proteomic analysis of TGF-β1 treated HPMCs

HPMCs were treated with 1 ng/mL TGF-β1 for 48 and 96 h. Harvested samples were mixed with buffer containing 9.5 M urea, 2% Triton X-100, 5% β-mercaptoethanol, 1 mM PMSF, 5 μg/mL aprotinin, 10 μg/mL pepstatin A, 10 μg/mL leupeptin, 1 mM EDTA, 10 mM Na_3_VO_4_ and 10 mM NaF. Protein concentration was quantified using Bio-Rad assay and loaded onto two-dimensional SDS-PAGE and protein spots were detected with silver staining. 2D-gel images were scanned using an image scanner (Amersham Biosciences) and quantitatively analyzed using ImageMaster software in duplicate each sample. Differentially expressed protein spots (87 spots) were picked and identified by MALDI-TOF-MS.

#### Sample preparation for mass spectrometric analysis

Samples were prepared described previously^60^. Briefly, spots were excised with a scalpel, destained by 15 mM K_4_FeCN_6_/50 mM sodium thiosulfate, and washed. The gels were dehydrated by acetonitrile, rehydrated by adding 10–20 μL of 25 mM NH_4_HCO_3_ with 20 ng/μL sequencing-grade trypsin (Promega, WI, USA), and incubated at 37 °C for 15–17 h. The extracts were pooled and evaporated to dryness in Speedvac for mass spectrometric analysis.

#### Mass spectrometric analysis

The dried peptides were mixed with saturated matrix solution (a-cyano-4-hydroxycinnamic acid in 60% acetonitrile-0.1% trifluoroacetic acid) and analyzed with matrix-assisted laser desorption ionization-time of flight mass spectrometry (MALDI-TOF-MS) (Voyager-DE STR; Applied Biosystems, Inc.) or electrospray ionization quadrupole-time of flight tandem mass spectrometry (ESI-q-TOF MS/MS, Micromass/Waters Corp.). Using the Micromass ProteinLynx Global Server (PLGS) 2.1 data processing software, the data output was shown as a single MASCOT-searchable peak list (.pkl) file. The peak list files were used to query the SwissProt database using MASCOT (global search engine). All reported assignments were verified by automatic and manual interpretation of spectra from Mascot in a blinded mode.

#### Evaluation of differentially expressed proteins

Among the proteins identified through MALDI-TOF-MS, we picked differentially expressed proteins and focused on proteins with at least a 1.5-fold change at one time point (48 and/or 96 h).

### Construction of functional network (EMT-network) for DEGs

A functional network of DEGs elicited during TGF-β1 induced EMT, was constructed based on a known probabilistic human functional gene network^22^. A total of 16,243 genes and 476,399 linkages were downloaded with evidences from HumanNet (http://www.functionalnet.org/humannet/). The network construction was performed using Cytoscape. Differentially co-expressed genes in at least two microarray datasets were used as a seed set. The initial network based on the seed set was extended to first neighbors in order to comprise genes not showing significant differences in expression but with an indirect relation to the DEGs. Overall, the EMT-network was composed of seed nodes and first neighbor nodes, and interactions between selected nodes. Additionally, genes with consistent expression patterns in the proteome data were marked in a distinctive manner in the EMT-network.

### Detection of dense network clusters by topological properties

To detect highly interconnected clusters within the EMT-network, topological properties were analyzed by using Molecular COmplex Detection (MCODE) algorithm^61^. We used the MCODE v1.4.1 plugin in Cytoscape 3.3.0. To detect dense network clusters, we repeated the analysis by changing the node score cutoff (NSC), which is the most essential parameter for defining the cluster size in the growing module^62^. We performed a total of 10 experiments by parameters keeping degree cutoff = 2, without loop inclusion, K-core = 2, and with haircut on. Eight different conditions for the node score cutoff (0.4, 0.3, 0.2, 0.1, 0.05, 0.025, 0.01, and 0.005) were tested with the following parameters keeping max depth = 100 and no fluff. We tested two more experiments by keeping the node score cutoff as the default, 0.2, and changing max depth = 50 or with fluff being turned on.

### Prioritization of network clusters enriched with DEGs

For clusters identified from 10 independent analytical runs with different MCODE parameters, the hypergeometric test was performed to prioritize dense network sub-modules with enriched DEGs under the assumption that a network sub-module with enriched DEGs represents an essential functional module for developing TGF-β1-induced EMT. For up- and down-regulated DEGs, sub-modules with more than 3 nodes and *P* values less than 0.01 were selected. To consider candidates for *in vitro* evaluation, we took note of sub-modules enriched with DEGs in experiments using HPMCs. Also, we further examined sub-networks with *P* value less than 0.05 if those are expanded from previously selected sub-modules or marked with differentially expressed proteins (DEPs).

### Small-interfering RNA transfection and adenoviral transduction of HPMCs

Human TNFAIP6 and ZC3H12A small-interfering RNA (siRNA) and non-targeting siRNA as a negative control (Dharmacon, Inc., Chicago, IL) were used at 20 nmol/L. Opti-MEM transfection media and lipofectamine (both from Invitrogen, Paisley, UK) were used for transfection. HPMCs were seeded 1 day before transfection and cultured to 40–50% confluence on the following day. RNA interference (RNAi) duplexes for target genes were mixed with lipofectamine to form a transfection complex that was added to cells in 6-well plates. At 24 hours after knockdown of target genes, cells were incubated in M199 medium containing 1% FBS for 24 h and then with or without 2 ng/mL TGF-β1 for 48 h.

The recombinant adenovirus NNT (Ad-NNT) was custom generated by Vector Biolabs (Philadelphia, PA). In brief, human NNT1 was cloned into E1- and E3- shuttle plasmids, and used to generate [E1-E3-] Ad vectors. Ad vectors were generated by inserting CMV-driven GFP. Green fluorescent protein adenovirus (Ad-GFP) (Seoulin Corp., Seoul, Korea) was used as a negative control. HPMCs were infected with NNT (Ad-NNT) and Ad-GFP at a concentration of 50 and 100 multiplicity of infection (MOI) for 24 h. After transfection, the cells were incubated in M199 medium containing 1% FBS for 24 h, then further incubated with or without 2 ng/mL TGF-β1 for 48 h.

### Real-time PCR

Gene expression was assessed by real-time PCR using SYBR Green method (ABI 7300 real-time PCR thermal cycler, Applied Biosystems, Foster City, CA, USA). The gene expressions were normalized to GAPDH levels. The sequences of the primer pairs were as follows: αSMA, forward 5′-TCC GGA GCG CAA ATA CTC TGT-3′ and reverse 5′-CCG GCT TCA TCG TAT TCC TGT-3′; Fibronectin, forward 5′-CCA AGA AGG GCT CGT GTG A-3′ and reverse 5′-GGC TGG AAC GGC ATC AAC-3′; and E-cadherin, forward 5′-GGC CTG AAG TGA CTC GTA ACG A-3′ and reverse 5′-CAG CCG CTT TCA GAT TTT CAT C-3′; and ZO-1, forward 5′-GAA TAT GAA AAG GAA TCT CCC TAT GG-3′ and reverse 5′-AGC AAG CCA AGA GCC TT-3′ and TNFAIP6, forward 5′- GAA GCA CGG TCT GGC AAA TAC-3′and reverse 5′-CTG CCT CTA GCT GCT TGT AAG TTG-3′; and ZC3H12A, forward 5′-CAG ATG AGC TCC GTG CCA AT-3′and reverse 5′-CAG AGA GCT GGA CTG GGA TGA-3′; and NNT, forward 5′-GCT GAC ATG CCC GTC GTT AT-3′and reverse 5′-CCA CGA TGG TCA GCA GAT TG-3′; and GAPDH, forward 5′-TTC ACC ACC ATG GAG AAG GCT-3′and reverse 5′-TGG TTC ACA CCC ATG ACG AAC-3′.

### Statistical analysis

Data are expressed as mean ± SE. Mean values obtained from each group were compared using ANOVA with subsequent post hoc tests. *P* value of < 0.05 was used as criterion for statistical significance.

## Supplementary information

Supplementary Figures

Supplementary Tables

## Data Availability

The datasets used and/or analyzed during the current study available from the corresponding author on reasonable request.
