# Supplementary material for: Network-based integrated analysis of omics data reveal novel players of TGF-β1-induced EMT in human peritoneal mesothelial cells
Source: Sci Rep. 2019 Feb 6;9:1497. doi: 10.1038/s41598-018-37101-9 (PMC6365569; doi:10.1038/s41598-018-37101-9)

Network-based integrated analysis of omics data reveal novel players of TGF-β1-induced EMT in human peritoneal mesothelial cells

Soo Min Han[1,2], Hye-Myung Ryu[3], Jin-Joo Suh[1], Kong-Joo Lee[1], Soon-Youn Choi[3], Sangdun Choi[4], Yong-Lim Kim[3*], Joo Young Huh[5*], Hunjoo Ha[1]

Supplementary Figure 1. Gene set enrichment analysis of reactome pathways of up-regulated genes.

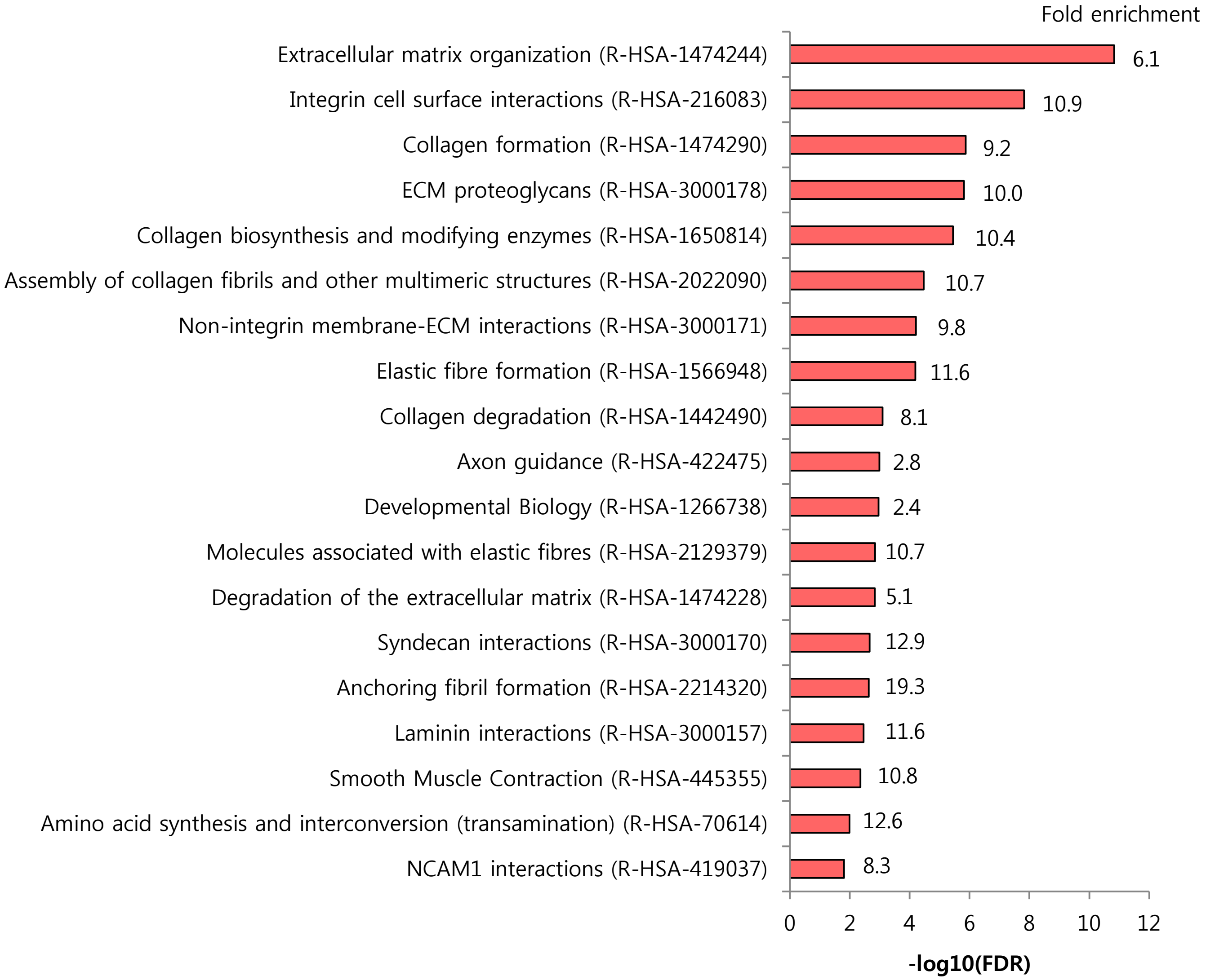

Supplementary Figure 2. 2D maps of protein expression profiles, representing differently expressed proteins (DEPs) by TGF-β1 treatment. Control HPMC cells (a,c) and TGF-β1 treated HPCMs for 48h (b) and 96 h (d).

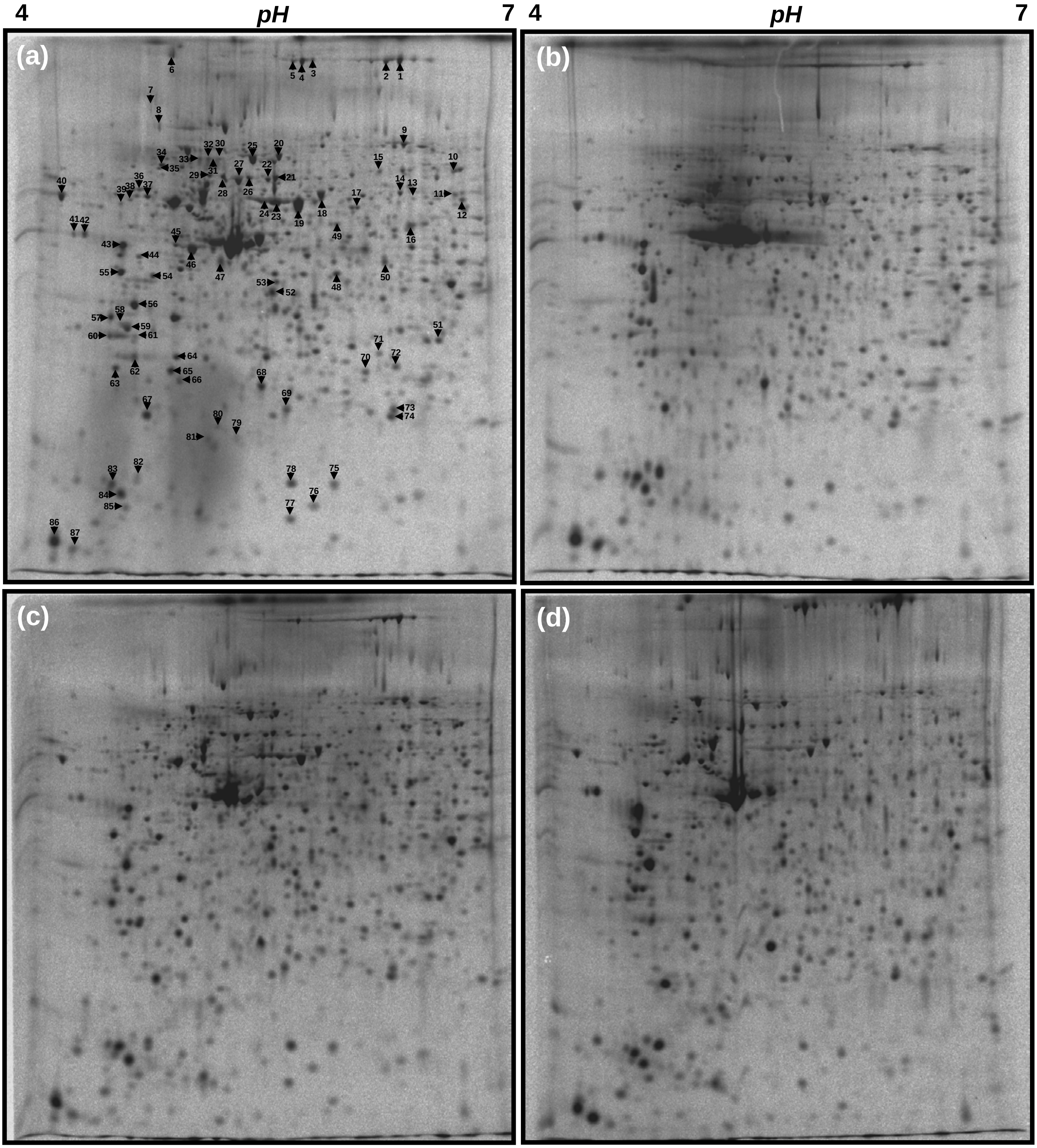

2D gel electrophoresis images of newly synthesized proteins after TGF beta treatment. Control HPMC cells(A,C) were labeled with 2. 5μCi/ml ^{35}S methionine in methionine free DMEM media(GIBCO O472) for 1hr. HPMC cells were treated with 2ng/mL TGF -beta for 48hr(B) , 96hr(D). And then pulse labeled ^{35}S methionine for 1hr. Same amounts of protein samples were separated on 2D gel electrophoresis and autoradiographed by BAS2500.

Supplementary Figure 3. Proteomic analysis. A) Functional classification of 93 proteins showing differential expression by TGF-β1 treatment in HPMCs. B) The hierarchical tree of GO MF terms overrepresented by DEPs (FDR < 0.05). Node colors and size represent the statistical significance of functional enrichment of the corresponding GO MF terms, drawn using BinGo. The white nodes were not statistically significant (FDR > 0.05), but shown to link downstream overrepresented nodes.

A

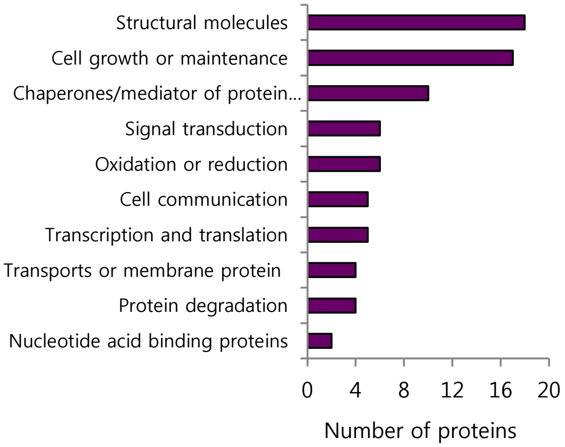

B

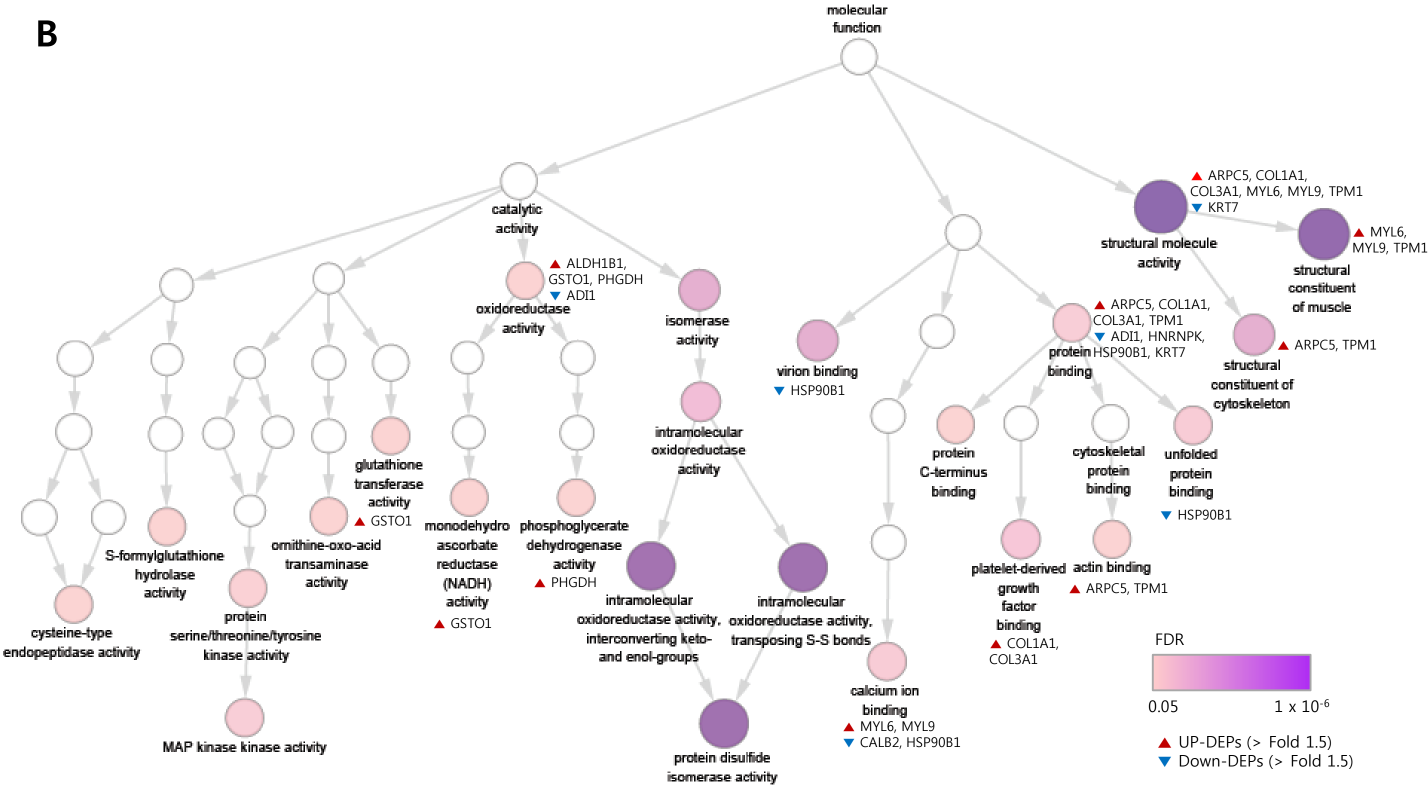

Supplementary Figure 4. Venn diagram of overlapped genes between different datasets.

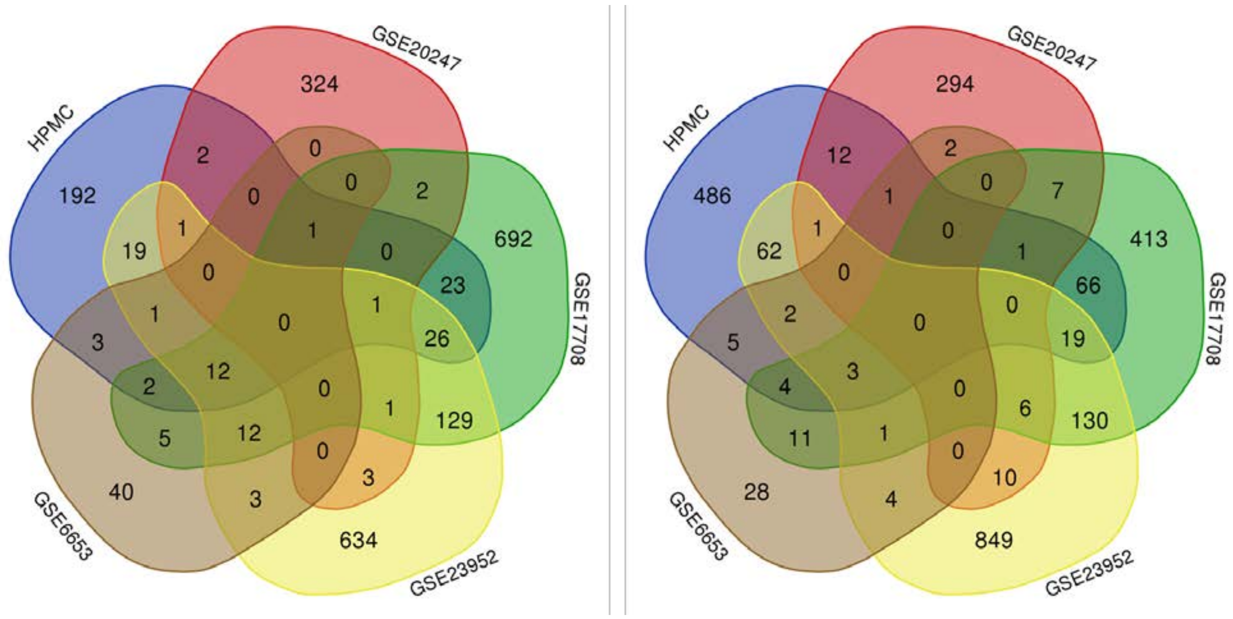

Up-regulated DEGs **Down-regulated DEGs**

Supplementary Figure 5. The transfection efficiency of *TNFAIP6* and *ZC3H12A* knockdown and *NNT* overexpression. Values are means ± SE of 4 experiments. *p<0.05 vs control

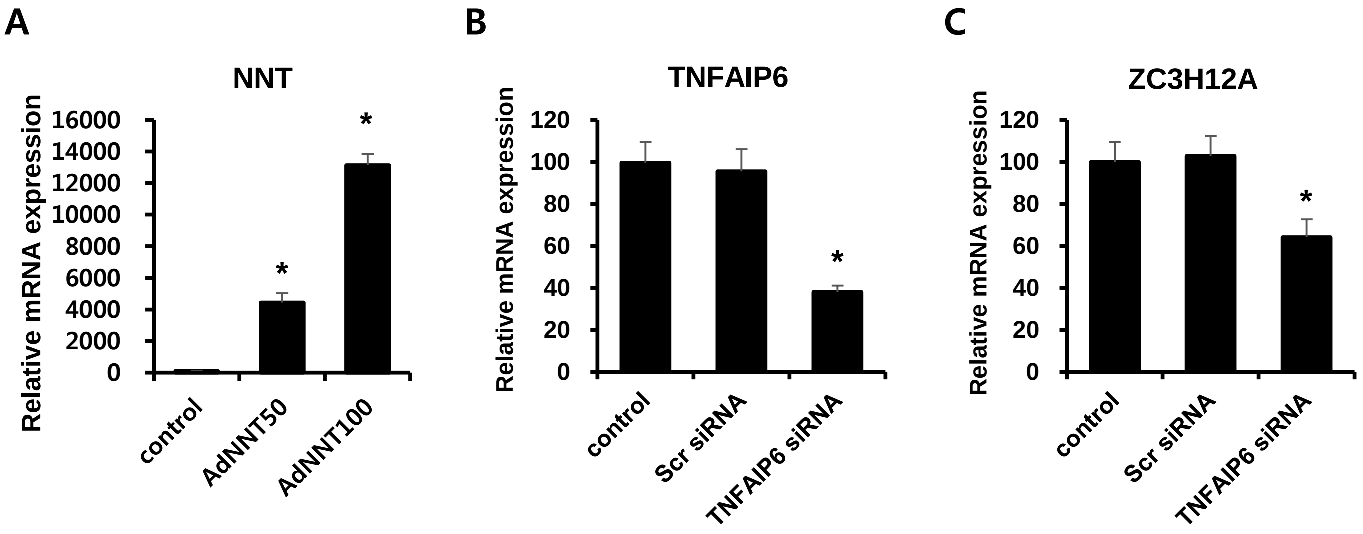

Supplement: Supplementary file 1 — Supplementary Figures [file 41598_2018_37101_MOESM1_ESM.pdf]
